# Clonal Occurrence of *Salmonella* Weltevreden in Cultured Shrimp in the Mekong Delta, Vietnam

**DOI:** 10.1371/journal.pone.0134252

**Published:** 2015-07-29

**Authors:** Gazi Md. Noor Uddin, Marianne Halberg Larsen, Lisa Barco, Tran Minh Phu, Anders Dalsgaard

**Affiliations:** 1 Department of Veterinary Disease Biology, Faculty of Health and Medical Sciences, University of Copenhagen, Copenhagen, Denmark; 2 World Organization for Animal Health (OIE), National Reference Laboratory for Salmonella, Istituto Zooprofilattico Sperimentale delle Venezie, Legnaro (Padua), Italy; 3 College of Aquaculture & Fisheries, Can Tho University, Can Tho City, Vietnam; University of Aveiro, PORTUGAL

## Abstract

This study investigated the occurrence, serovar and antimicrobial resistance of *Salmonella* spp. in shrimp samples from intensive and extensive farms located in three different provinces in the Mekong Delta, Vietnam. Shrimp from 11 of the 48 farms all contained *S*. Weltevreden, except for one farm yielding *S*. Agona, with no difference in *Salmonella* occurrence between the two production systems. Pulsed field gel electrophoresis (PFGE) of *S*. Weltevreden showed closely related *Xba*I pulse types, suggesting a clonal relationship despite the farms and shrimp samples being epidemiologically unrelated. *S*. Weltevreden was susceptible to most antimicrobials tested, with a few strains being resistant to florfenicol, chloramphenicol, sulfamethoxazole or trimethoprim. Future studies of the ecology of *S*. Weltevreden should establish if this serovar may survive better and even multiply in warm-water shrimp farm environments compared to other *Salmonella* serovars.

## Introduction

*Salmonella* spp. is one of the most common causes of food-borne infections worldwide. Poultry meat and eggs are predominant reservoirs of *Salmonella* spp. in many countries, but other animal meats such as pork, beef and fish are also important vehicles of infections [1]. In recent years, there has been increasing interest in the water-borne aspect of this pathogen, due to several outbreaks associated with the consumption of *Salmonella* spp.-contaminated drinking water [2] and a frequent occurrence in vegetables and certain seafood products [3–6]. In Vietnamese catfish, a *Salmonella* spp. prevalence as high as 50% has been reported [7], but in general a prevalence of up to 20% may be found in shrimp [3, 8–10]. In general a higher occurrence of *Salmonella* spp. has been reported in seafood products originating from Asian and African countries compared to products from Europe and North America [10–11].

The natural reservoir and ecology of *Salmonella* serovars associated with aquatic environments are not fully understood. *Salmonella* spp. are primarily inhabitants of the gastrointestinal tract of mainly warm-blooded animals and reptiles [11]. *Salmonella* spp. typically reaches the aquatic environment via fecal contamination and may be able to survive for months or even years in the aquatic environment after fecal shedding [11–12]. However, *Salmonella* serovars isolated from seafood are often different from those found in the gastrointestinal tract of animals, including birds [5, 11], indicating other reservoirs. The main serovars found in seafood and tropical waters include *S*. Weltevreden, *S*. Senftenberg, *S*. Rissen, *S*. Lexington, *S*. Saintpaul, *S*. Newport, *S*. Albany and *S*. Anatum [3, 8, 10, 13–17]. Specific sources of *Salmonella* in aquaculture may include both livestock and non-livestock-associated reservoirs, such as fecal-contaminated pond water, fish feed, birds, amphibians and reptiles [18–20]. The aim of this study was to determine the occurrence, genetic diversity and antimicrobial resistance of *Salmonella enterica* serovars isolated from cultured shrimp in the Mekong Delta, Vietnam. Samples were taken from intensive and extensive farms in order to compare the findings between the two production systems.

## Methods

### Study sites and sampling of shrimp

Farmers collect shrimp in plastic baskets from nets when ponds are harvested, and sort them by size using their bare hands. The shrimp are then weighed, placed in large plastic containers with ice, and transported by truck to common collection sites where different farmers deliver their shrimp, before being taken to processing factories in the Mekong Delta, Vietnam. Shrimp culture in extensive farms is practiced with natural feed and water exchanges provided by the tide and without the use of chemicals, while intensive shrimp farms exchange water everyday by pumping, and use commercial pelleted feed and chemicals to control water quality and disease. According to a survey that mapped shrimp culture areas in the Mekong Delta, Vietnam [21], the Bac Lieu, Ca Mau and Soc Trang provinces are main culture areas of black tiger and white leg shrimp intensive aquaculture. Extensive shrimp farms (16 farms) were selected in An Minh District, Kien Giang province which together with Ca Mau province are main locations of extensive shrimp culture. However, extensive shrimp farms were not selected in Ca Mau province as these farms increasingly are located close to intensive farms. Seventeen black tiger and 15 white leg (*Littopenaeus vannamei*) shrimp samples from intensive farms in Soc Trang and Bac Lieu provinces (one sample per farm) were also collected. Specific authority permission or ethical clearance is not required according to Vietnamese regulations for collection of cultured shrimp. Shrimp samples were collected at shrimp collection sites before being delivered to processing plants. We assure that the field studies did not involve endangered or protected species. The three provinces are the main shrimp producers in the Mekong Delta, Vietnam. Each sample was collected at the common collection sites and consisted of five shrimp (20 to 30 g/shrimp) originating from one farm. All shrimp samples were collected in sterile plastic bags, kept in an insulated box with ice (no direct contact between the shrimp and ice), and transported to the laboratory where they were analyzed within 24 hours of sampling.

### Isolation of *Salmonella* from shrimp

The exoskeleton, including the head, of the shrimp was removed using sterile scissors. Meat, including intestines, from five shrimp (100–150 g) originating from one farm were pooled as a representative sample for one farm and then transferred into a sterile stomacher bag with 1 ml of buffered peptone water (1% w/v) and manually homogenized using a pestle and mortar. *Salmonella* was isolated in 10 g of the homogenate per sample according to the ISO 6579 protocol [22]. Up to five presumptive *Salmonella* colonies on Xylose-Lysine Deoxycholate plates (XLD; Oxoid, Basingstoke, Hampshire, England) were subcultured onto Tryptic soy agar plates (Merck, Germany) and confirmed by a traditional slide agglutination test using anti-*Salmonella* polyvalent sera (Statens Serum Institut, Copenhagen, Denmark) and by genus-specific PCR [23]. Isolates were stored at -80°C in Brain Heart Infusion broth (Oxoid) with 30% glycerol. All isolates positive in the agglutination test and PCR were further characterised by sero- and PFGE typing.

### Serotyping

*Salmonella* serotyping was performed according to the White–Kauffmann-Le Minor scheme [24] by slide agglutination with O- and H-antigen specific sera (Statens Serum Institute, Copenhagen, Denmark) at the World Organization for Animal Health (OIE) and Italian National Reference Laboratory for Salmonellosis, Istituto Zooprofilattico Sperimentale delle Venezie, Padua, Italy.

### Pulsed field gel electrophoresis (PFGE) and PFGE pattern analysis

PFGE genotyping was performed to determine the genetic diversity and epidemiological association of *Salmonella* isolated from different geographical areas and shrimp production systems. PFGE genotyping was undertaken according to the CDC PulseNet protocol following DNA digestion with *Xba*I [25] to establish the genetic variation and relatedness between isolates. *S*. Braenderup H9812 was used as a reference strain [26]. Analysis of the DNA fingerprints was performed using GelCompar II (version 4.6) software (Applied Maths, Belgium) and a Dice coefficient with a band position tolerance of 1% and 0.5% optimisation level was applied. The clustering was performed by the unweighted pair group method with arithmetic averages (UPGMA) to produce a dendrogram with banding patterns evaluated according to the criteria suggested by Tenover et al. [27].

### Antimicrobial susceptibility testing

**Fig 1:**
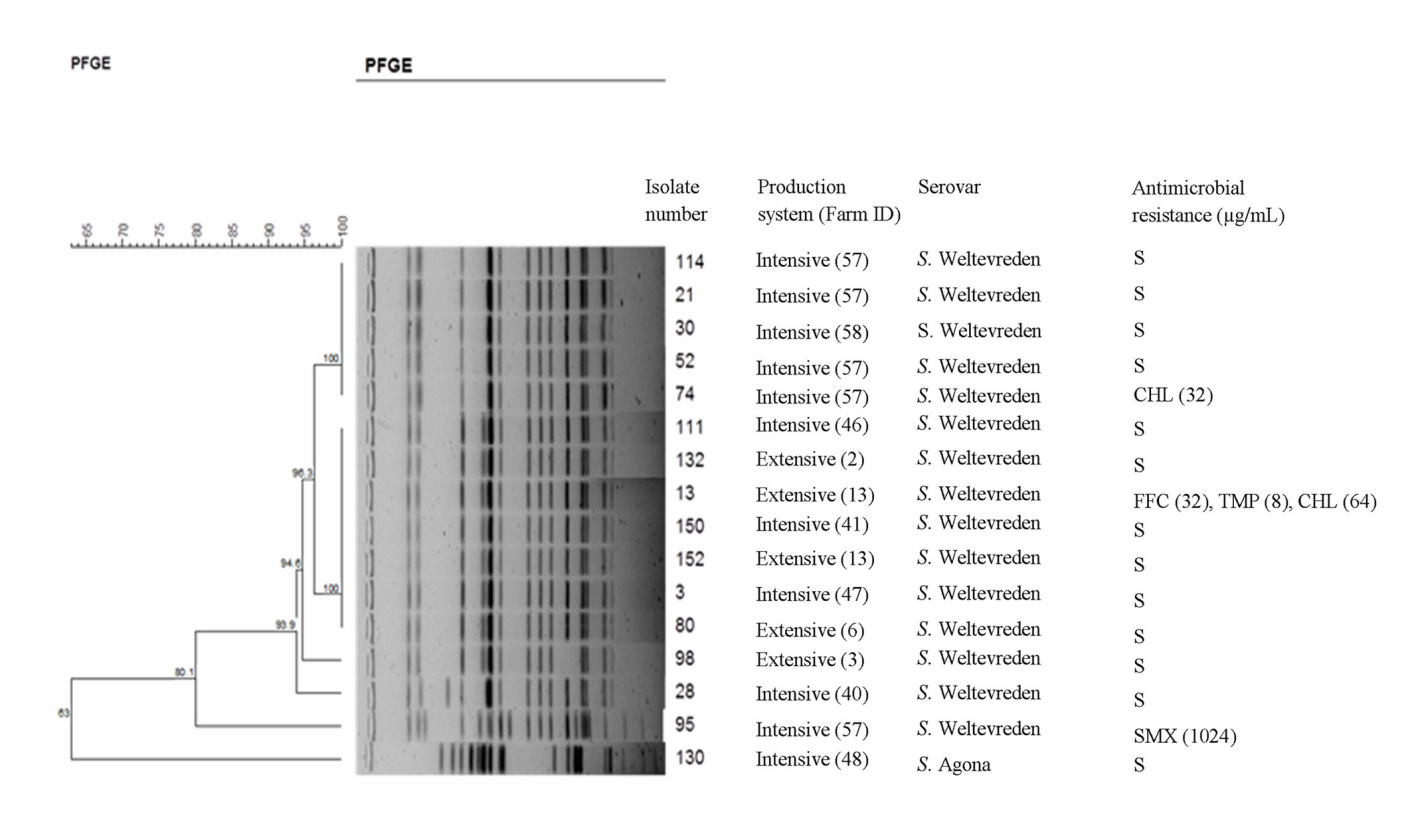
*Xba*I PFGE patterns and antimicrobial susceptibility of *Salmonella* isolates from shrimp samples from intensive and extensive production system. Similarity analysis was performed using the Dice coefficient, and clustering was done by the unweighted pair-group method with arithmetic averages (UPGMA; position tolerance 1.0%). Antimicrobial resistance was determined using the Sensititre Vizion System and MIC values were evaluated in accordance with the protocol of the Clinical and Laboratory Standards Institute. FFC, Florfenicol; CHL, Chloramphenicol; SMX, Sulfamethoxazole and TMP, Trimethoprim; "S" means susceptible to all antimicrobials tested. MIC values (μg/mL) are shown in brackets.

*S*. Weltevreden (17 isolates) and *S*. Agona (1 isolate) representing different PFGE types and at least one isolate per shrimp sample (farm) (Fig 1) were selected for antimicrobial susceptibility testing using Sensititre Vizion System (Trek Diagnostics System, East Grinstead, UK) according to the protocol of the Clinical and Laboratory Standards Institute [28]. The 17 *S*. Weltevreden isolates selected included two isolates showing a blurred PFGE banding pattern as well as four isolates with an identical pulse type from farm 57 and two isolates with an identical pulse type from farm 13 (Fig 1). The antimicrobial susceptibility of these farm isolates were tested to determine if isolates from the same sample and with identical pulse type showed identical susceptibility patterns. Tests were performed for β-lactam (ampicillin, AMP (0.25 μg/mL); cefotaxime, CTX (8 μg/mL); ceftazidime, CAZ (8 μg/mL)) and non-β-lactam antimicrobials (chloramphenicol, CHL (8 μg/mL); gentamycin, GEN (4 μg/mL); tetracycline, TET (4 μg/mL); sulfamethoxazole, SMX (256 μg/mL); trimethoprim, TMP (2 μg/mL); streptomycin, STR (no standard breakpoints available); colistin, CST (2 μg/mL, for *Pseudomonas aeruginosa*); florfenicol, FFC (4 μg/mL, for *S*. *choleraesuis*); kanamycin, KAN (16 μg/mL); nalidixic acid, NAL (16 μg/mL) and ciprofloxacin, CIP (9.06 μg/mL)). Standard breakpoints for *Enterobacteriaceae* for most of the antimicrobials except for florfenicol and colistin were used [29]. Standard breakpoints of FFC and CST were used respectively for *Salmonella choleraesuis* and *Pseudomonas aeruginosa* [29].

## Results

A total of four of the 16 shrimp samples from extensive farms and seven of the 32 samples from intensive farms contained *Salmonella*, with no clear difference in the occurrence of *Salmonella* between the two production systems or shrimp species (Table 1). The serovar of 51/55 isolates were determined and further characterised by PFGE analysis. *S*. Weltevreden (with the antigenic formula 3:r:z6) were isolated from all *Salmonella*-positive extensive and intensive farms, except for one intensive farm which was found positive for *S*. Agona (antigenic formula 4,12: f,g,s:-) only.

**Table 1:** Prevalence of *Salmonella* in extensive and intensive cultured shrimp in the Mekong Delta, Vietnam.

Province	Farm type/shrimp species	No. of *Salmonella* positive farms
Kien Giang	Extensive/ *P*. *monodon*	4/16
Soc Trang & Bac Lieu	Intensive/ *P*. *monodon*	2/17
Soc Trang & Bac Lieu	Intensive/ *L*. *vannamei*	5/15

PFGE typing showed that all 47 *S*. Weltevreden isolates were closely related, suggesting a clonal relationship. Isolates from four extensive and three intensive farms showed an identical pulse type (Fig 1). There did not seem to be any association between pulse type, geographical location and production system. One shrimp sample (farm 57) contained *S*. Weltevreden isolates with two different pulse types. PFGE typing of four *S*. Agona strains recovered from one sample originating from one intensive farm revealed an identical pulse type that was clearly different from types found for *S*. Weltevreden (Fig 1).

Susceptibility to 14 antimicrobials was tested on the 18 isolates shown in the Fig Antimicrobial resistance to FFC were shown by three *S*. Weltevreden isolates (32 μg/mL) and one isolate was intermediate resistant (8 μg/mL), but all isolates were sensitive to most other antimicrobials tested (Fig 1). Among four isolates from one intensive farm (no. 57) with an identical pulse type, one isolate was resistant to CHL, whereas the others were fully sensitive. Similarly, two isolates from farm 13 showing an identical pulse type showed different susceptibility patterns with one isolate from an extensive farm being resistant to FFC, CHL and TMP indicating that isolates with an identical pulse type can show different phenotypic antimicrobial susceptibility pattern. Six isolates from extensive and intensive farms showed an identical pulse type and were fully susceptible. Two *S*. Weltevreden isolates showing resistance to CHL and FFC and TMP and FFC respectively are not listed in the Fig 1 since repeated PFGE analysis produced blurred band fragments. *S*. Agona isolates showed an identical pulse type and were resistant to FFC only (32 μg/mL).

## Discussion

*Salmonella* Weltevreden strains were isolated at a similar prevalence from extensive (4/16) and intensive (7/32) cultured shrimp farms located in three different provinces in the Mekong Delta, Vietnam (Table 1). Although samples from more shrimp farms may have been included in the study to strengthen the comparison of *Salmonella* prevalence in the two production systems, the farms selected are representative of extensive and intensive shrimp culture as practiced in the Mekong Delta. The occurrence of *S*. Weltevreden with closely related or identical pulse type(s) and similar antimicrobial susceptibility patterns (Fig 1) from epidemiologically unrelated farms suggests a clonal relationship. This could indicate a common faecal source, *e*.*g*. wastewater discharge, feed or animals such as birds, reptiles and rodents that continuously contribute *S*. Weltevreden. Another plausible hypothesis is that this serovar may survive better and even multiply in warm-water shrimp farm environments compared to other *Salmonella* serovars. Although untreated wastewater is discharged from different types of urban areas located in the three provinces, it is unlikely that *S*. Weltevreden originates from wastewater or fecally-contaminated surface run-off water as humans, livestock and other animals mainly can be expected to harbor and excrete other serovar like *S*. Typhimurium and *S*. Enteritidis [5]. However, *S*. Weltevreden has been reported to be a common serovar isolated from humans in Vietnam, Thailand and Malaysia [3, 6, 30–31] and is also detected in cattle, pig and poultry in Vietnam [30]. It has been known for years that livestock feed is an important source of *Salmonella* and that the pathogen may also constitute a hazard in aquaculture feeds [18–19]. However, in contrast to intensive shrimp farms, the extensive farms do not use artificial feed, but depend on natural feed organisms present in the coastal waters where shrimp culture is practiced. The large distances, *i*.*e*. 60–80 km, between the studied shrimp farms in the three provinces suggest that a common animal reservoir is an unlikely source of *S*. Weltevreden. Follow-up studies are needed to elucidate if specific waterfowl species are commonly seen at shrimp farms and if such birds may be a potential common source of *S*. Weltevreden The shrimp samples analyzed were obtained from common collection sites, where they were delivered from the individual farms after the shrimp were harvested, collected in plastic baskets, sorted by bare hands, and transported in plastic containers with ice. Although some level of fecal contamination may occur during this chain of events, *e*.*g*. contaminated ice, the shrimp from the different farms were handled and sampled separately, *e*.*g*. different baskets, sources of ice and transport containers were used, making it unlikely that the collected shrimp had been contaminated with *S*. Weltevreden from a common source.

The closely related or identical *XbaI* PFGE genotypes could also be due to a general low genetic heterogeneity in *S*. Weltevreden. However, Thong et al. [31] report that 95 clinical and environmental *S*. Weltevreden strains show 39 distinct *XbaI* PFGE profiles with a wide range of Dice coefficients (0.27 to 1.00), indicating some genetic heterogeneity and that multiple clones exist. This is supported by Ponce et al. [32], who genotyped 37 *S*. Weltevreden strains isolated from seafood, mainly originating from Asia, imported into the USA from 2001 to 2005. They found four different *XbaI* PFGE clusters with a genetic similarity of 66% to 76% representing different sources, countries and years of isolation. Further studies should document the genetic relatedness of *S*. Weltevreden isolated from different sources, including humans, in the Mekong Delta.

Except for one intensive shrimp farm that yielded *S*. Agona, *S*. Weltevreden was the only serovar isolated. *S*. Weltevreden seems to occur with higher frequencies in seafood, in particular from Asia, than other *Salmonella* serovars as compared with Europe and the USA, where *S*. Enteritidis and *S*. Typhimurium are mainly found in foods but also in patients with gastroenteritis. Koonse et al. [33] analyzed 1,234 samples from 103 shrimp farms in six countries (including three Asian countries) in 2001 to 2003 and found *S*. Weltevreden to be the most frequently isolated serovar, accounting for 21% of the total number of serovars isolated. *S*. Weltevreden was also the frequently isolated serovar (17.6%) among 210 *Salmonella* strains isolated from seafood imported into the USA from 2001 to 2005 [33] and among 27 different serovars isolated in different types of fresh and raw seafood samples (n = 417) in Cochin, India [8]. A review of the trends in serovars of *Salmonella* causing infections in humans, and occurring in food and water in Thailand between 1993 to 2002, showed that out of a total of 118 serovars identified in 44,087 clinical samples analyzed, *S*. Weltevreden accounted for between 7.9% and 13.5% of all serovars seen annually [6]. The review also found *S*. Weltevreden in 26.3% (265/1007) of frozen seafood samples. Human gastroenteritis in Malaysia has also increasingly been associated with *S*. Weltevreden, accounting for 31% of all serovars identified between 1989 and 1994 [34]. Seafood-associated *Salmonella* infection has also been reported in India [8], Hong Kong [12] and Spain [35]. Despite *S*. Weltevreden being the most common serovar isolated in imported seafood in Europe and the USA, the prevalence in seafood appears low compared with *Salmonella* in other types of food, *e*.*g*. poultry, eggs and pork, and food safety risks due to *Salmonella* seem low for consumers of imported seafood, which is probably in contrast to seafood consumers in Asia. Altogether, the different reports of *S*. Weltevreden being the most commonly isolated *Salmonella* serovar in seafood, but also in water, aquatic plants and ducks, suggest a water-related source [6]. In their extensive study of *Salmonella* in shrimp farms, Koonse et al. [33] isolated *S*. Weltevreden more often than other serovars and also found the occurrence of *Salmonella* associated with water-related sources, such as pond water, pond sediment and processing water. However, they also found a significant (*P* = 0.0342) relationship between the log number of fecal bacteria (both fecal coliforms and *E*. *coli*) and the probability that any given sample would contain *Salmonella* [33]. They therefore suggest that *Salmonella* is not part of the natural flora of the shrimp culture environment and is not inherently present in shrimp grow-out ponds.

*S*. Weltevreden and *S*. Agona were susceptible to most antimicrobials tested, with a few *S*. Weltevreden strains being resistant to CHL, FFC, SMX or TMP (Fig 1). Four identical pulse-type isolates from one intensive farm (no. 57), of which one isolate was CHL resistant, showed a pulse type closely related to a type shown by another *S*. Weltevreden from the same farm, which was resistant to SMX (Fig 1). The generally high level of antimicrobial susceptibility of *S*. Weltevreden corroborates other studies of clinical, environmental and food isolates [32, 36– 37] and may indicate an environmental reservoir of the *S*. Weltevreden isolated in this study [20]. Future molecular studies of *S*. Weltevreden should determine factors that may be associated with increased survival in aquatic environments, and whether the CHL and FFC resistances found are related to the *flo*R gene conferring CHL and FFC resistance in *S*. Typhimurium DT104 [38].

A report from an FAO expert workshop reviewed the occurrence of *Salmonella* in aquatic environments and seafood, and documented a significantly higher occurrence in tropical compared to temperate aquatic environments [11]. As dynamics of contamination with *Salmonella* in aquatic environments may be associated with specific seasonal patterns or climate characteristics, more frequent storm-generated flows and torrential rains (*e*.*g*. monsoon) in tropical areas that transport *Salmonella* from original source points to the sea *via* aquifers, streams and rivers may account for the higher *Salmonella* occurrence in tropical environments [39]. On the other hand, intense sunlight and high water temperatures are critical factors expected to determine a more rapid reduction of *Salmonella* in tropical waters. However, warm waters together with high levels of organic matter and typical conditions prevailing in tropical areas may contribute to a more appropriate habitat for an increased survival of *Salmonella* [11]. The ability to form biofilm on a wide variety of contact surfaces may allow *Salmonella* to persist for several months outside animal hosts, *e*.*g*. an *in vitro* study found that *S*. Weltevreden has a biofilm-forming ability on plastic, cement and steel surfaces [40]. However, there is a requirement for in-depth ecological studies of *Salmonella* in aquatic environments that document how *S*. Weltevreden and other serovars survive and possible multiply. Such *in vivo* studies should establish the conditions under which *Salmonella* serovars may multiply in tropical aquaculture. The *S*. Weltevreden isolated in the current study would provide good candidate strains for such experiments, which may be conducted with strains inoculated into microcosmos placed in shrimp ponds.
